# Combination of Zinc Oxide Photocatalysis with Membrane Filtration for Surface Water Disinfection

**DOI:** 10.3390/membranes13010056

**Published:** 2023-01-02

**Authors:** Santiago Martínez Sosa, Rosa Huertas, Vanessa Jorge Pereira

**Affiliations:** 1 iBET—Instituto de Biologia Experimental e Tecnológica, Apartado 12, 2780-901 Oeiras, Portugal; 2 LAQV-REQUIMTE, Departamento de Química, Faculdade de Ciências e Tecnologia, Universidade NOVA de Lisboa, 2829-516 Caparica, Portugal; 3 Instituto de Tecnologia Química e Biológica António Xavier, Universidade NOVA de Lisboa, Av. da República, 2780-157 Oeiras, Portugal

**Keywords:** membrane processes, ZnO nanoparticles, photocatalysis, water disinfection

## Abstract

Increase water usage has led to its deterioration. Pollutants are easily found in the aquatic environment and treatment techniques must keep improving to meet the current needs and future demands. Membranes are attractive for water treatment, but limitations like fouling and the highly concentrate produced affect their performance. Combining membrane filtration with photocatalysis provides the opportunity to integrate a self-cleaning step during membrane filtration. In this work, we studied two simple and efficient approaches to combine membrane filtration with zinc oxide nanoparticles (using the catalyst in suspension and immobilized) activated by light emitting diodes (LED) emitting light at 365 nm. Both systems were used to test the disinfection efficiency in real surface water, compared in terms of catalyst concentration in the permeate stream (below the limit of detection) and its recovery after filtration (higher that 74%). The system’s capability to retain and inactivate target bacteria (total coliforms and *E. coli*) in the retentate stream was tested with samples of real surface water. The results obtained show that both configurations led to an improved performance in comparison to the membrane treatment alone with a higher retention of the bacteria (not detected in the permeate samples) and higher treatment of the retentate. For the modified membranes, different catalyst concentrations and thermal treatments were tested. The performance of all the processes was evaluated in terms of the level of treatment achieved and the permeate flux. All the modified membranes showed an efficient retention of the target bacteria from surface water, with higher performances than the unmodified membrane (96.2% for total coliforms and 94.9% for *E. coli*). Remarkable retention and treatment of the retentate was achieved using a membrane modified with a catalyst load of 125 mg subject during two hours to a thermal treatment of 300 °C. This modification has a performance comparable to the system with the same catalyst load in suspension. During operation, the permeate flux reduction is lower with the modified membranes which could lead to longer operation times without the need of further cleaning or replacement. The combined system, ceramic membranes modified with zinc oxide and UV-A LEDs proved to be effective to retain and disinfect water quality indicator bacteria present in real surface water matrices.

## 1. Introduction

Water is essential for several activities including the support of the biosphere [1]. However, the interconnection between its consumption and its use affects its quantity and quality [2]. Agriculture consumes 70% of global water withdrawals [3], industrial processes and energy generation compete for the rest of the available water, using it for non-consumptive applications [4,5,6]. If proper treatment is not applied, these activities will introduce pollutants such as pathogens, heavy metals and chemicals in the water sources, leading to a resource that may not meet appropriate quality standards [6,7]. To evaluate water quality, indicators such as total coliforms and *E. coli* are routinely monitored. The presence of *E. coli* in water samples is associated to possible fecal contamination by humans or animals. Effective treatment will help prevent contamination before reaching the final use. Thus, treatment techniques must keep improving to meet the challenges that new pollutants, more stringent regulations, climate change and water demand will bring looking to prevent future shortages and outbreaks [5,8,9,10].

Pressure driven membrane treatment processes are extremely promising to upgrade conventional treatments and ensure the production of high-quality potable water. However, the retention of pollutants on the membranes leads to the production of a concentrated retentate and a decline in the permeate flux over time [11,12,13]. Modification of the membranes with nanomaterials can help reduce fouling by altering membrane characteristics. Inorganic ceramic membranes should be used as substrates for modifications, due to their higher stability to prevent damage and ensure a longer treatment process performance [14,15,16]. Silicon carbide membranes in particular show exceptionally hydrophilicity, low transmembrane pressure increase due to low fouling and high removal of non-organic matter [17]. These promising characteristics make them especially attractive for water treatment [17,18,19,20,21,22].

The combination of photocatalytic nanoparticles with membrane processes represents an opportunity to integrate a self-cleaning step into membrane operation. Pollutants can be degraded to innocuous substances through advanced oxidation processes (AOPs) [23,24,25,26]. The development of photocatalytic membranes represents a promising improvement, as it tackles problems of the individual technologies [27,28,29,30,31]. Even though several studies focused on the combinations of photocatalysis with membrane processes [32,33,34,35], the application of photocatalytic membranes in industry is still limited and publications are mostly focused on titanium dioxide (TiO_2_) modifications, while it has been reported that zinc oxide (ZnO) has a higher photocatalytic performance for the degradation of organic pollutants [36,37,38]. Additionally, most of the studies focus on the treatment of model solutions (e.g., methylene blue), which don’t capture the complexity of a real water matrix composition and therefore the results can’t predict the water treatment process effectiveness under real conditions. Future industrial interest in the application of modified ZnO photocatalysts is expected due to its nontoxicity, photostability, high photocatalytic activity, and consequent ability to degrade chemical pollutants that may affect the environment, marine life and human health such as organic dyes, phenolic compounds and persistent organic pollutants [39]. The photocatalytic nanoparticles can be combined with membrane filtration when used in suspension or immobilized in the membranes. Suspended systems usually achieve superior treatment due to the large surface area of photocatalyst offered by the suspended particles. However, the necessity of separation and recovery of the catalyst in the retentate stream represent an increase in the overall complexity of the process. Immobilized systems overcome these steps by attaching the catalyst to the membrane’s surface. However, the efficiency of this approach strongly depends on the catalyst load, its distribution on the membrane and the immobilization methodology.

In this study, composite photocatalytic membranes were produced by immobilizing ZnO nanoparticles on the surface of silicon carbide (SiC) inorganic membranes, chosen due to their inherent chemical and thermal resistance. Variations on the catalyst concentration and thermal treatment of the modified membranes were applied to evaluate their effect on the membrane’s performance. The performance of the modified membranes was also compared with the results obtained using an unmodified control and unmodified membranes with the catalyst in suspension. This work allowed us to increase the knowledge on photocatalytic membrane processes and the conditions that can improve the performance of this technology. Specifically, this work is focused on the development of SiC photocatalytic membranes, using preformed commercial ZnO materials. The simple method proposed can also add value to the existing knowledge and favor steps towards their application at industrial level. Moreover, using real water samples also helped us to understand the potential of these systems in conditions closer to a real water treatment plant.

## 2. Materials and Methods

### 2.1. Photocatalytic Membrane System

The photocatalytic membrane system used included a filtration system with a capacity of 250 mL where the untreated surface wastewater (UTSW) was deposited, with an UV-LED device coupled on top of the sample container (Figure S1 in the Supplementary Materials section). The UV-LED device used was a triple wavelength system (AquiSense Technologies, Erlanger, KY, USA) with a triple LED UVinaireTM and a control box. The UV-LED device was placed 7 cm above the membrane compartment. A wavelength of 365 nm was used as this UV-A wavelength is not absorbed by the ozone layer and can thus reach the earth’s surface. The membranes (modified and unmodified) were placed in the membrane compartment of the system and a sterile Schott flask was placed below to collect the permeate sample. The system was connected to a vacuum pump GAST-DOA-P504 (GAST, Benton Harbor, MI, USA).

### 2.2. Nanoparticles and Membrane Modification

ZnO nanoparticles were purchased from Sigma-Aldrich, Saint Louis, MO, USA (Product Number: 544906) with a particle size below 100 nm (71 nm) and a specific surface area of 15 m^2^/g (Batch Number MKCM4723). Different suspensions were prepared with 0.25, 0.5 and 1 g/L of ZnO powder dispersed in deionized water (DIW) and stirred at 25 °C for two hours. A NanoZetaSizer particle size analyzer (ZS 90, Malvern, Worcestershire, UK) was used for the analysis of the size distribution of the ZnO nanoparticles in suspension. The size distribution was studied using samples from the ZnO suspension with a concentration of 0.5 g/L. Three samples were prepared and analyzed in triplicate. The characterization was made at a temperature of 20 °C, with an equilibrium time of 50 s. Three measurements were taken within 10 s each. The ZetaSizer Sofware 7.1 software (Malvern, Worcestershire, UK) was used to collect and analyze the data. Although the ZnO nanoparticles used have a diameter below 100 nm, all the samples showed a similar mean diameter of about 446 nm due to the aggregation of the nanoparticles in suspension, a phenomenon that has been reported by other authors [40,41].

Circular SiC discs with a diameter of 47 mm were cut from commercial flat sheet ultrafiltration membranes (LiqTech International, Ballerup, Denmark). All the membranes were cleaned before use with diluted citric acid (Panreac, Barcelona, Spain) with a 2% *w*/*v* concentration for 24 h [42], followed by cleaning with DIW and a thermal treatment at 100 °C for one hour. After cleaning, the membranes were further modified through a solvent-free process using ZnO suspensions with the initially prepared suspensions and dried. The membranes were installed in the filtration system and modified through physical deposition of the nanoparticles at room temperature with a vacuum of 0.1 bar. The ZnO layers deposited over the membrane substrates had a homogeneous appearance (Figure S2 in the Supplementary Materials section). The first batch of membranes was modified using the ZnO suspension of 0.5 g/L and then dried at 100 °C in an oven (Memmert UM 300, Schwabach, Germany) for 24 h, leading to membranes modified with 125 mg of ZnO. The second batch with ZnO suspensions of different concentrations (0.25 g/L, 0.5 g/L and 1 g/L) and then dried at 100 °C in the oven for 24 h, producing membranes modified with 62.5, 125 and 250 mg of ZnO, respectively. Finally, the last batch of membranes was modified with a suspension of 0.5 g/L and then dried at different temperatures (300, 500, 700 °C; heating rate of 250 °C/h) for two hours in a muffle (Nabertherm furnace LT 9/14, Lilienthal, Germany) producing again membranes modified with 125 mg of ZnO. According to the commercial specifications, the temperatures tested did not damage the ceramic support.

The morphology of the membranes was characterized using a field emission gun scanning electron microscope (FEG-SEM) by JEOL (Tokyo, Japan) model JSM7001F, equipped with a light elements energy dispersive spectroscopy detector (EDS) by Oxford Instruments (England), model INCA 250, operating at an accelerating voltage of 25 kV. Four different magnifications were used to analyze the top surface of the membranes and one (200×) to analyze the cross section. The EDS analysis identified the elements present in the membrane. Three different points of each membrane were selected for the SEM images. The microscope images were analyzed using the ImageJ-FIJI software [43,44,45]. This software has been previously used by other authors and compared to different established characterization methods, showing that most membrane properties obtained by image analysis of micrographs were within acceptable accuracy [46,47,48,49,50]. This analysis is only valid for surface pores as it was done in the present work. To measure the particle size of the images, the “morphological segmentation” plugin from ImageJ-FIJI was used. The process followed to analyze the micrographs and the equations used are available in the Supplementary Materials Section S3–S5.

### 2.3. Safety and Recovery of Catalyst in Different Configurations

To verify that the catalyst could be recovered after operation in the assays conducted with the catalyst in suspension and was not present in the permeate samples, four different assays were conducted:

(1) Recovery of catalyst in 250 mL of suspension (0.5 g/L);
(2) Recovery of catalyst in 250 mL of suspension (0.5 g/L) with the UV-LED light emitting at 365 nm;
(3) Stability of catalyst in the modified membrane after filtration of 250 mL of DI water (DIW);
(4) Stability of catalyst in the modified membrane after filtration of 250 mL of DI water with the UV-LED light emitting at 365 nm.

Before each experiment, all the unmodified membranes, modified membranes and the nanoparticles used for the suspension assays were weighted. In the first two experiments conducted in suspension, after filtration the ZnO particles were removed from the membrane with DIW. This physical washing allowed the recovery of the catalyst and its reuse in the following repetitions. After five repetitions, the final suspension was dried in a heating plate at 50 °C until the water was removed, then the weight of the recovered ZnO measured at room temperature was compared to the weight measured at the beginning of the experiment to prepare the initial suspension. The weight comparison showed the amount of catalyst recovered after the experiments.

The third and fourth experiments were performed with the modified membrane with the catalyst immobilized on the surface (250 mg). Here, 250 mL of DIW were filtered with or without using the UV-LED system. The experiments were also repeated five times. After, all the modified membranes were dried in a vacuum desiccator and weighted to measure if any catalyst was lost through the operation of the system.

Moreover, to verify if the nanoparticles reached the permeate, the permeate samples were analyzed in terms of their Zn content by inductively coupled plasma atomic emission spectroscopy (ICP-AES). For this evaluation, seven calibration solutions (0, 0.05, 0.10, 0.25, 0.50, 0.75 and 1.00 mg/L) were prepared to build a calibration curve. This curve was used to calculate the concentration of Zn in the permeate samples.

### 2.4. Treatment Performance of Real Surface Water Samples

To test the proposed treatment system, several real surface water samples were collected from the Tagus River between April and July 2021. The sampling point selected was Algés beach at the municipality of Oeiras, Portugal. Samples were collected during high tide in 20 L containers and stored at 4 °C until use. All the treatment experiments were carried out one day after the collection of the samples to preserve their properties and without applying any chemical-physical adjustment.

To evaluate the disinfection performance, the photocatalytic-membrane system was tested by filtering 250 mL of UTSW. For the experiments with the catalyst in suspension, 125 mg of ZnO were suspended in 250 mL of UTSW (0.5 g/L) right before starting the test. This concentration is equivalent to the catalyst load of 125 mg used to modify the membranes. All the experiments were carried out in the same conditions (25 °C and a vacuum pressure of 0.1 bar). In total, seventeen different assays were performed (Table 1). To have samples of all streams, after 180 mL of sample were filtered, the system was stopped and samples of the feed, permeate and retentate were collected.

Two controls were performed: the first one, a sample of the UTSW kept under the laboratory visible light and the second one, a sample of the same UTSW kept in dark. Controls helped to evaluate the effect of visible light on the samples, no relevant differences in bacteria concentration were found when the controls were compared with the feed samples.

### 2.5. Quantification of Total Coliforms and Escherichia coli in Water Samples

To test the concentration of total coliforms and *E. coli* in the samples the IDEXX Colilert-18 analysis test was used. Three dilutions of each sample were prepared (1:1, 1:10, and 1:100). Dilutions were mixed with a chemical substrate with indicators o-nitrophenyl-β-D-galactopyranoside (ONPG) and 4-methylumbelliferyl-β-glucuronide (MUG) to detect the bacteria. Finally, the samples were poured into quanti-tray containers, sealed and incubated at 35 °C for 18 h. After incubation, the trays were examined under visible and UV-light (Model UVL-21) emitting at 366 nm. The positive wells were counted, and the results were evaluated with the IDEXX most probable number (MPN) generator software. These results were used to calculate the percent rejection of each target microorganism, the percent treatment. In addition, the permeate flux through the membrane was monitored during the different assays.

## 3. Results

### 3.1. Recovery of Catalyst

The results obtained in the assays conducted to test the recovery of the catalyst after filtration are summarized in Table 2.

Higher recovery of ZnO was obtained from the membranes with the ZnO layer (higher that 91%) compared with the recovery of ZnO nanoparticles in suspension (higher that 73%). The higher recovery of catalyst from the membranes with the ZnO layer is attributed to the extensive procedure required to recover the catalyst in suspension as nanoparticles were in contact with several containers, leading to some loss of particles which should also be considered in large scale processes.

The concentration of zinc in the permeate samples was measured by inductively coupled plasma atomic emission spectroscopy. The permeate samples from the experiments with the modified membranes showed a lower concentration of zinc when compared to the system where the catalyst was used in suspension (Table 3). A rejection of 99.89% or higher was obtained in all the assays. Despite of some differences in Zn concentration between the samples, all samples showed a concentration significantly below the 5 mg/L limit established by the EPA for drinking water to avoid a metallic taste [51]. From the results, there is no evidence that the UV-LED light has an important effect to on the amount of catalyst that permeates through the membrane.

### 3.2. Treatment Performance

**Figure 1:**
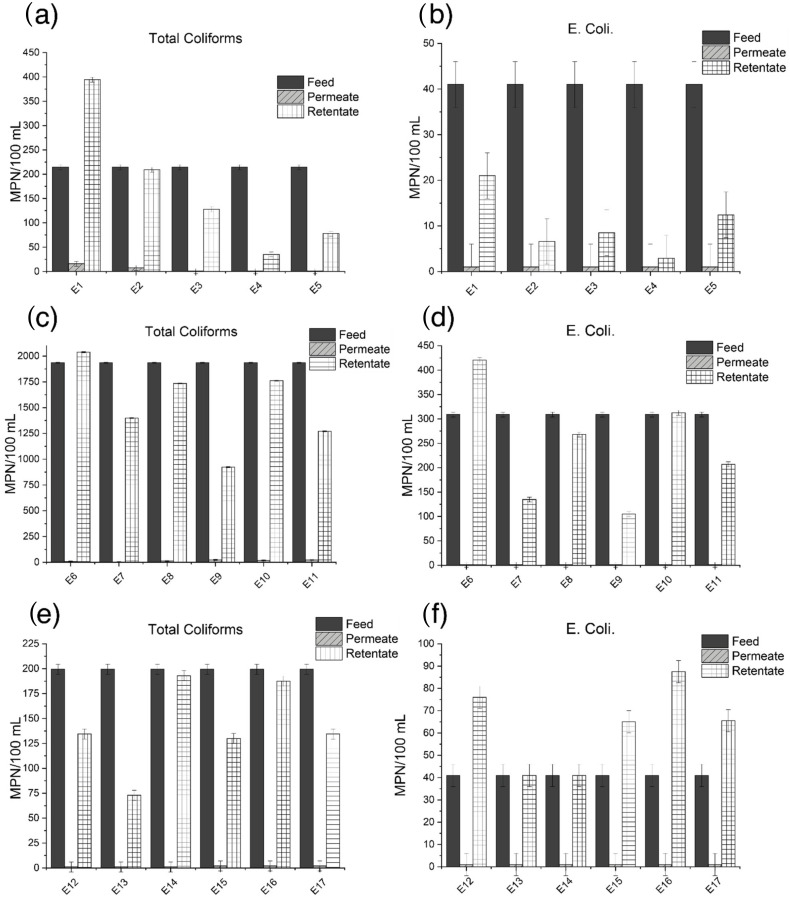
Concentration of bacteria on each stream after the filtration (**a**) Total coliforms E 1–5 (**b**) *E. coli* E 1–5 (**c**) Total coliforms E 6–11 (**d**) *E. coli* E 6–11 (**e**) Total coliforms E 12–17 (**f**) *E. coli* E 12–17. Most of permeates had a concentration of bacteria below the detection limit of the analysis (1 MPN/mL).

The IDEXX Colilert-18/Quanti-Tray 2000 test was used to evaluate the efficiency of the membranes to treat bacteria in different conditions. The results of the concentration of total coliforms and *E. coli* on each stream after the different experiments are presented in Figure 1.

To compare the performance of the photocatalytic membrane systems the rejection to each bacteria and the treatment performance were also calculated (Table 4).

The first comparison between the unmodified membrane (E 1–2), the system with catalyst in suspension (E 3–4) and the first membrane with ZnO immobilized (E 5) showed that the combined membrane-photocatalytic system achieved rejections higher than 99.6% for total coliforms and 97.6% for *E. coli*, and treatment percentages of 99.0% for total coliforms and *E. coli* when the catalyst was used in suspension. This superior performance of the system with the catalyst in suspension has been previously reported comparing TiO_2_ in different configurations [42] and its performance is related to the higher effective surface area of the particles in suspension. Nevertheless, the results with the catalyst immobilized (E 5) also showed a concentration in the permeate samples below the detection limit, showing the potential of the system. Even though the treatment performance of the immobilized system (E 5) is lower than the suspended system (E 4), the concentration of bacteria in the immobilized system is still reduced compared to the unmodified membrane. The difference in performance is associated to the reduction of the specific surface area of the catalyst immobilized on the membrane.

The second experiment comparing the effect of the amount of catalyst used to modify the membranes showed a clear difference between modified membranes. In the experiments without UV light the retentate treatment was lower. However, they showed a high rejection, indicating that the modification changed the characteristics of the pore size, thus enhancing the concentration of bacteria in the retentate. When the UV light was included, the concentration of bacteria was reduced, showing the best treatment performance with the membrane with a catalyst load of 125 mg (E 9). This is associated to a combination of a homogeneous catalyst layer and an optimum amount of catalyst, able to produce enough reactive oxygen species (ROS) for the treatment of bacteria. The results agree with other studies in terms of agglomeration and surface area of the catalyst [42]. Higher concentrations of catalyst do not lead to better treatment, as agglomeration of particles would reduce the available surface area of catalyst. Also, the morphology of the layer has an important effect on the performance of the system. Thus, a homogeneous layer of catalyst will impact positively the final performance of the photocatalytic layer.

The last experiment evaluated the changes in the surface produced by thermal treatment after modification with a catalyst load of 125 mg. Results show no relevant advantage on thermally treating the modified membranes at higher temperatures in terms of better treatment of bacteria. Although the experiments show a good retention for both bacteria, treating the modified membranes at temperatures higher than 300 °C affect the layer of catalyst, resulting in defects on the surface. These imperfections on the ZnO layers may have reduced the adherence of the catalyst to the membranes, resulting in the loss of catalyst or areas where microorganisms can grow without being affected by the UV-light, leading to fouling. Mendes et al. [52] tested the antibacterial action and target mechanisms of zinc oxide nanoparticles against the clinically relevant bacteria *Escherichia coli*, *Staphylococcus aureus*, *Pseudomonas aeruginosa*, and the Gram-positive model *Bacillus subtilis*. Seventy percent of the cells exhibited damage in the cytoplasmic membrane after 15 min of exposure to the ZnO nanoparticles. The authors concluded that ZnO nanoparticles affect different structures of the bacteria cells, but their main mechanism of action is in the cytoplasmic membrane, being other structure effects a consequence or secondary effect of the membrane rupture [52]. To extend the optical absorption of ZnO from UV to visible light region, Pant et al. [53] proposed a combination of silver and zinc oxide carbon fiber composites that exhibited a strong photocatalytic and antibacterial activities.

### 3.3. Impact of Catalyst Load on the Permeate Flux

**Figure 2:**
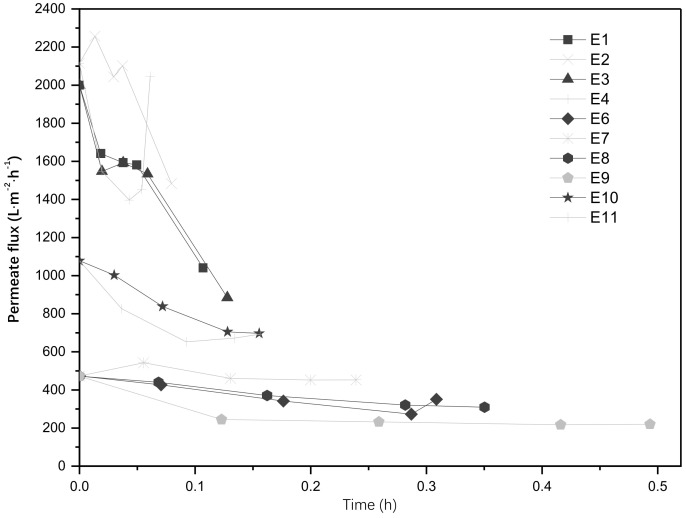
Permeate flux comparison between membranes with different catalyst loads.

During the experiments presented in Section 3.2, the permeate flux through the membrane was also evaluated. Results comparing the unmodified membrane, the system with the catalyst in suspension and the membranes modified with different loads of catalyst are shown in Figure 2.

**Figure 3:**
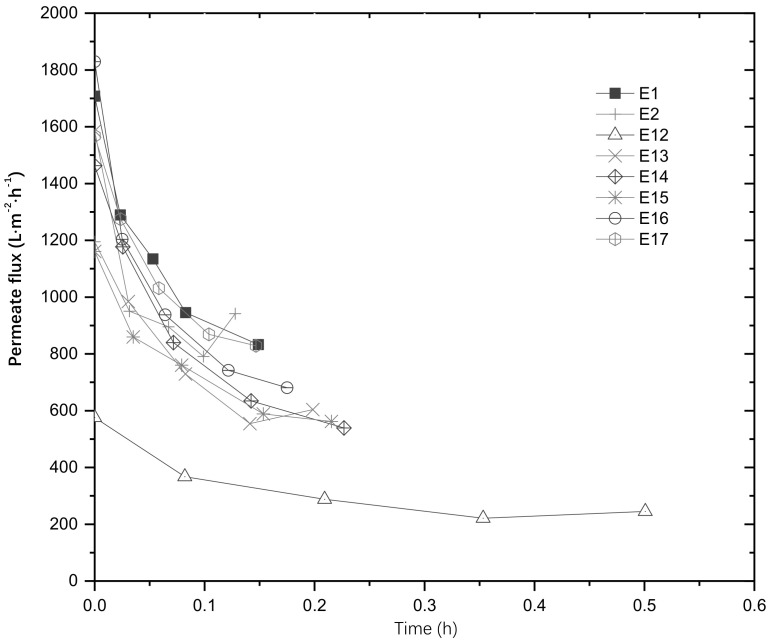
Permeate flux comparison between membranes modified with 125 mg of ZnO and treated at different temperatures.

The results comparing the membranes with different loads of catalyst show an increase in the permeate flux with the use of the UV light, which is associated with an increase in the hydrophilicity of the SiC membrane when exposed to UV light. If the bacteria are hydrophobic and the hydrophilicity of the unmodified SiC membrane is increased, there are less chances for the bacteria to attach to the membrane, thus they have more chances to be exposed to UV light, increasing the treatment of the retentate. The decrease on the permeate flux with time is attributed to the adsorption of pollutants to the surface of the membrane (fouling), increasing its resistance to mass transport. When the catalyst was used in suspension the fast decrease in flux was attributed to the presence of the ZnO particles in the feed, although the ZnO nanoparticles are not considered foulants per se, they affect the permeate flux when they deposit on the surface of the membrane. With the modified membranes there is an initial reduction on the permeate flux, indicating that the catalyst layer modified the characteristics of the membrane surface. However, through the operation of the system, the permeate flux is almost constant, only reducing by 4% with respect to its initial flux. This demonstrates the benefits that the combined system could represent for water filtration, as a constant permeate flux would also increase the operating time of the system. Figure 3 shows the permeate flux comparing the unmodified membrane with the membranes modified and treated at different temperatures. For the membranes treated at high temperatures, the drop in flux was not as significant as in the previous experiments, which may be associated with the morphology of the membranes which showed defects after treatment.

### 3.4. Characterization of the Membranes

The morphology of the top surface of the studied membranes was analyzed using SEM-EDS and Image J software, to identify differences of morphology, particle size, pore size and elemental distribution of the catalyst in the surface of the tested membranes. Table 5 shows the average thickness of the catalyst layer measured after the modifications. The deposition was clearly visible in the membranes due to the change in color (as shown in Figure S2 of the Supplementary Information section) but the change in thickness was not notorious as the thickness of the deposited layers (detailed in Table 5) was much lower than the thickness of the silicon carbide discs (1.9 mm).

**Figure 4:**
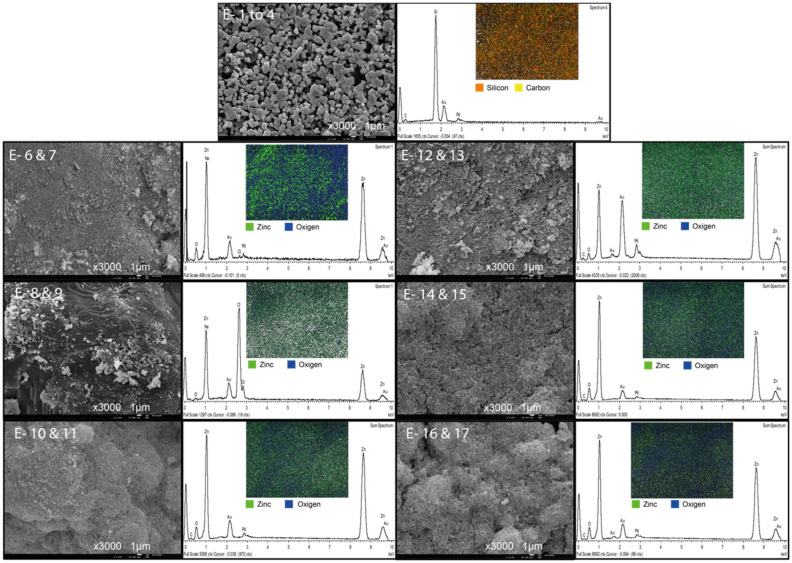
Catalyst distribution on modified membranes using EDS mapping made over SEM images.

Elemental analysis of the modified membranes is presented in Figure 4, showing the distribution of zinc and oxygen atoms on the surface of all the membranes.

The ZnO layer of all modified membranes showed a rough appearance since the nanoparticles tend to agglomerate. Lower catalyst deposited on the membrane surface led to a homogeneous surface, which explains the good rejection performance and treatment of the membranes modified with 125 mg of catalyst when compared to the unmodified membrane. The catalyst layer also justifies the reduction of permeate flux. Results show that increasing the catalyst load leads to the aggregation of nanoparticles which has been reported by other authors when high concentrations of nanoparticles are used [54,55]. These aggregates affect the adherence strength of the particles, producing loss of catalyst with further operation.

Thermal treatment of the modified membranes increased the defects on the ZnO layer which explains their lower treatment performance. The EDS analysis showed a homogeneous distribution of zinc and oxygen on the surface of all the membranes, but aggregates of particles are noticeable in the membranes with high catalyst concentrations and thermal treatment. This aggregates, support idea that high concentrations and high temperatures promote the formation of defects on the modified surface.

### 3.5. Membrane Surface Porosity and Particle Size

Different characteristics of the surface of the membrane were analyzed quantitatively using the microscope images and the software “Image J”. The results were arranged in a normal distribution to obtain the means of each parameter (Table 6).

The unmodified membrane has a much higher mean particle size than the modified membranes. The image analysis shows that all the surface of the modified membranes have a mean pore size around ten times smaller than the surface of the unmodified SiC membrane (E 1). The lower pore area at the surface of the modified membranes may explain the increase in the bacteria rejection performance and the decrease in the permeate flux of the modified membranes. However, if the defects of the ZnO layer are considered, the modification with nanoparticles leads to a heterogeneous layer with areas with a smaller pore size and high resistance and areas of low resistance, especially if the SiC layer is exposed. These exposed areas must be prevented, as they would lead to paths where the water not only has less resistance, but also there is no contact with the catalyst, thus preventing the photocatalytic treatment.

## 4. Conclusions

Photocatalytic membranes were successfully produced, characterized and evaluated in terms of their efficiency for the disinfection of real surface water samples.

The concentrations of zinc in the permeate samples were more than ten times lower than the limits established for drinking water. These results show that using ZnO nanoparticles combined with the tested commercially available SiC membranes are not expected to lead to a secondary pollution problem.

The filtration system with the catalyst in suspension and UV light showed a higher rejection/treatment performance than the system with the catalyst immobilized. However, the modified membranes with a catalyst load of 125 mg (75 g/m^2^) thermally treated at 300 °C after the modification, increased the rejection/treatment performance of the membrane. Also, these conditions lead to a homogeneous surface, resulting in a constant permeate flux behavior through operation. The modifications carried out suggest that higher concentrations of ZnO nanoparticles, as well as higher temperatures of thermal treatment don’t increase the rejection/treatment performance of the membrane. This is attributed to the aggregates of nanoparticles and defects on the catalyst layer found in the final product. Future studies should test the reusability of the modified membrane with the best performance in terms of retention, inactivation and permeability performance. This study provides further information for the development of photocatalytic membrane systems able to treat water.

## Figures and Tables

**Table 1:** Nomenclature used to characterize the different assays performed.

Sample ID	Membrane Type (ZnO_2_)	Thermal Treatment (°C)	Amount of ZnO (mg)	LED-UV Light
E-1	unmodified	No	0	No
E-2	unmodified	No	0	Yes
E-3	unmodified/ZnO_2_ in suspension (S)	No	125	No
E-4	unmodified/ZnO_2_ in suspension (S)	No	125	Yes
E-5	modified/ZnO_2_ in layer (L)	No	125	Yes
E-6	modified/ZnO_2_ in layer (L)	100	62.5	No
E-7	modified/ZnO_2_ in layer (L)	100	62.5	Yes
E-8	modified/ZnO_2_ in layer (L)	100	125	No
E-9	modified/ZnO_2_ in layer (L)	100	125	Yes
E-10	modified/ZnO_2_ in layer (L)	100	250	No
E-11	modified/ZnO_2_ in layer (L)	100	250	Yes
E-12	modified/ZnO_2_ in layer (L)	300	125	No
E-13	modified/ZnO_2_ in layer (L)	300	125	Yes
E-14	modified/ZnO_2_ in layer (L)	500	125	No
E-15	modified/ZnO_2_ in layer (L)	500	125	Yes
E-16	modified/ZnO_2_ in layer (L)	700	125	No
E-17	modified/ZnO_2_ in layer (L)	700	125	Yes

**Table 2:** Percentage of catalyst recovered after five filtration assays.

Experiment	ZnO Recovered (%)
SiC-ZnO (S)	73.5 ± 3.3
SiC-ZnO (S)-UV	78.4 ± 2.5
SiC-ZnO (L)	99.7 ± 0.2
SiC-ZnO (L)-UV	91.3 ± 0.2

**Table 3:** Concentration of Zn in the permeate samples collected after each filtration assay.

Characteristics	Concentration (mg/L)
Catalyst in suspension (S)	0.55
Catalyst in suspension (S) + UV	0.55
Catalyst in layer (L)	0.05
Catalyst in layer (L) + UV	0.06

**Table 4:** Percent rejection and treatment of each membrane for the target bacteria.

	Total Coliforms		*E. coli*	
Experiment	% Rejection	% Treatment	% Rejection	% Treatment
E1	92.6	70.9	97.6	89.3
E2	96.5	86.3	97.6	96.9
E3	99.5	91.9	>97.6	95.9
E4	>99.6	99.0	>97.6	99
E5	>99.6	97.0	>97.6	95.9
E6	99.5	69.3	>99.6	57.0
E7	>99.9	81.3	>99.6	87.8
E8	99.4	76.7	99.6	75.6
E9	98.8	87.6	>99.6	90.5
E10	99.1	76.3	99.7	71.6
E11	98.9	82.9	>99.6	81.2
E12	>99.5	87.5	>97.6	65.2
E13	>99.5	92.5	>97.6	79.2
E14	99.5	81.7	>97.6	80.8
E15	98.9	87.1	>97.6	68.3
E16	98.9	80.3	>97.6	54.7
E17	>99.5	87.0	>97.6	68.7

**Table 5:** Average thickness of ZnO layer obtained after the experiments.

Experiment	ZnO Layer Thickness (µm)
E 1–4	N/A
E 6–7	0.8 ± 0.1
E 8–9	26.4 ± 0.6
E 10–11	58.7 ± 1.3
E 12–13	31.3 ± 1.1
E 14–15	42.3 ± 0.2
E 16–17	31.6 ± 0.8

**Table 6:** ImageJ analysis of SEM images of the surface of the membranes.

Name	Membrane Surface					
	Mean Particle Size (nm)	Mean Pore Size (nm)	Pore Density (1/nm^2^)	Porosity (%)	Pore Circularity	Feret’s Diameter of Pores (nm)
E 1–4	484.4	371.8	1.1 × 10^−6^	17.5	0.6	546.6
E 6–7	42.8	38.7	1.3 × 10^−4^	24.7	0.7	54.9
E 8–9	34.9	56.7	3.4 × 10^−5^	16.7	0.7	81.9
E 10–11	61.8	49.7	4.8 × 10^−5^	10.2	0.5	62.3
E 12–13	59.1	41.9	6.2 × 10^−5^	14.7	0.7	61.7
E 14–15	53.0	41.3	9.0 × 10^−5^	20.1	0.7	60.2
E 16–17	60.7	48.6	4.8 × 10^−5^	14.4	0.6	70.8

## Data Availability

The data presented in this study are openly available in: https://doi.org/10.6084/m9.figshare.20527995.v1.

## Supplementary Materials

The following supporting information can be downloaded at: https://www.mdpi.com/article/10.3390/membranes13010056/s1, Figure S1: Schematic diagram of the filtration system used to test the unmodified and modified membranes. (1) UV-LED system (2) Feed container (3) Membrane filter (4) Permeate container (5) Vacuum pump. Figure S2: ZnO nanoparticles deposited on the modified membranes. Figure S3: (a) Division of the micrograph (b) Threshold adjustment (c) Normal distribution obtained with the values of pore size. Figure S4: (a) Division of the micrograph (b) Morphological segmentation (c) Threshold adjustment (d) Normal distribution obtained with the values of particle size. Figure S5: Cross section of the membranes modified and thermally treated.
