# Supplementary material for: Combination of Zinc Oxide Photocatalysis with Membrane Filtration for Surface Water Disinfection
Source: Membranes (Basel). 2023 Jan 2;13(1):56. doi: 10.3390/membranes13010056 (PMC9862052; doi:10.3390/membranes13010056)
Supplement: Supplementary file 1 [file membranes-13-00056-s001.zip › membranes-2000616-supplementary.pdf]

membranes

Supplementary Materials

Combination of Zinc Oxide Photocatalysis with Membrane Filtration for Surface Water Disinfection

Santiago Martínez Sosa [1,2], Rosa Huertas [1,2] and Vanessa Jorge Pereira [1,3,*]

[1] iBET-Instituto de Biologia Experimental e Tecnológica, Apartado 12, 2780-901 Oeiras, Portugal
[2] LAQV-REQUIMTE, Departamento de Química, Faculdade de Ciências e Tecnologia, Universidade NOVA de Lisboa, 2829-516 Caparica, Portugal
[3] Instituto de Tecnologia Química e Biológica António Xavier, Universidade Nova de Lisboa, Av. da República, 2780-157 Oeiras, Portugal
* Correspondence: vanessap@ibet.pt

S1. Photocatalytic membrane system used for the experiments.

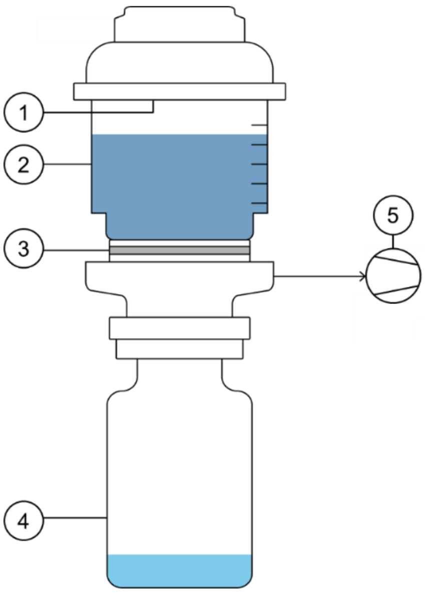

Figure S1. Schematic diagram of the filtration system used to test the unmodified and modified membranes. 1) UV-LED system 2) Feed container 3) Membrane filter 4) Permeate container 5) Vacuum pump.

S2. Membranes used in the experiments.

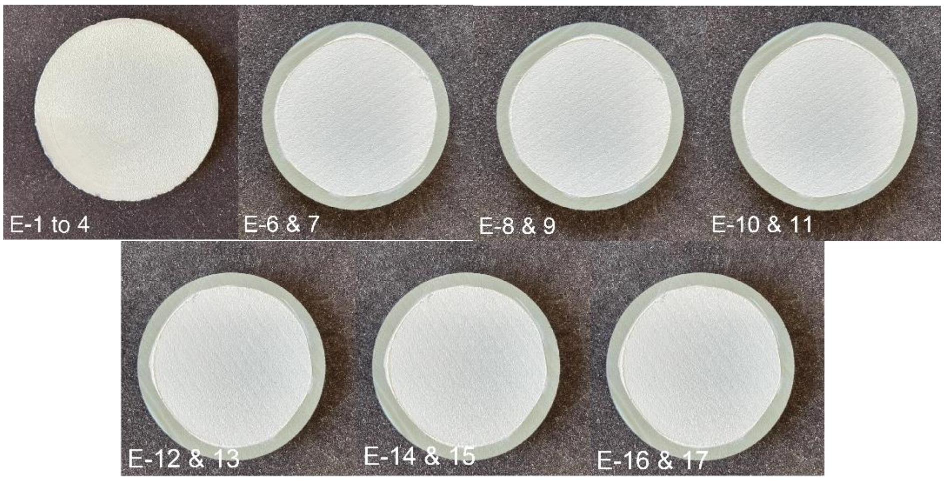

Figure S2. Visual appearance of unmodified membrane (E1 to 4) and the modified membranes (E6 to E17).

S3. Digital image processing.

All the SEM images were analyzed using the ImageJ-FIJI software (Version 1.53s) to evaluate porosity, pore density, mean pore size and mean particle size. For the unmodified membrane, the magnification of 3000× was selected, while for all the modified samples the magnification of 30,000× was selected.

To have an accurate measurement each image was divided in four different sections. First, the scale of the image was set using the scale of the micrograph, then the area for analysis was selected and the threshold of the image was adjusted. After filtering the binary SSimage the particles were analyzed, and the results were copied in a spreadsheet to evaluate the pore size using the area of the pores given by the software and equation 1 of the area of a circle. The process is illustrated in Figure S3.

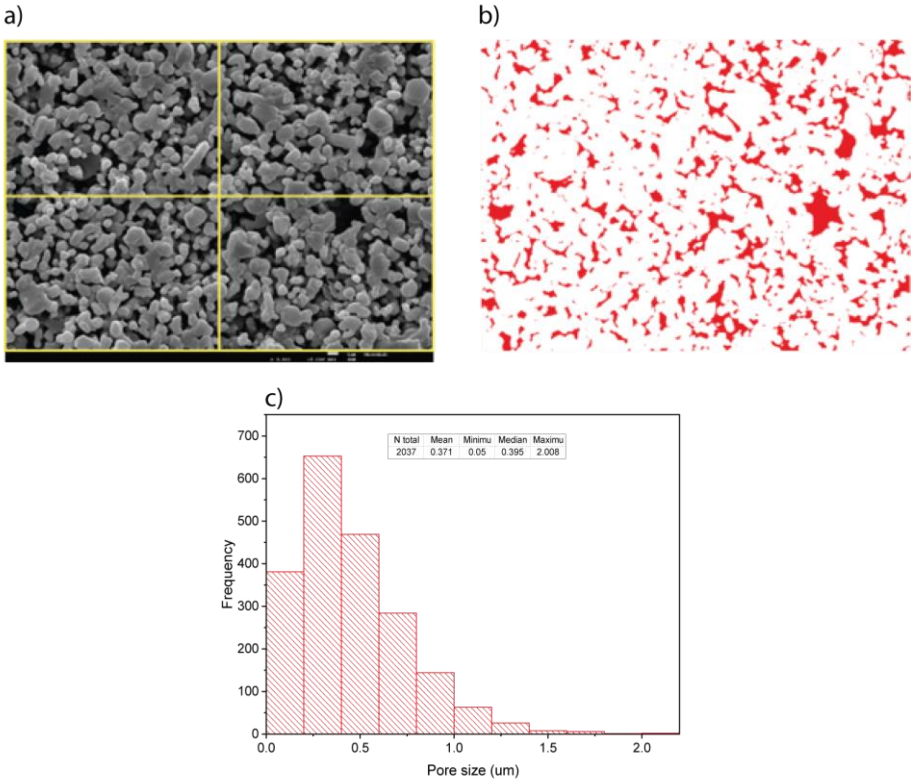

Figure S3. (a) Division of the micrograph (**b**) Threshold adjustment (**c**) Normal distribution obtained with the values of pore size.

For the analysis of the particle size a similar procedure was followed. However, the morphological segmentation plugin from ImageJ-FIJI was used to improve the identification of individual particles.

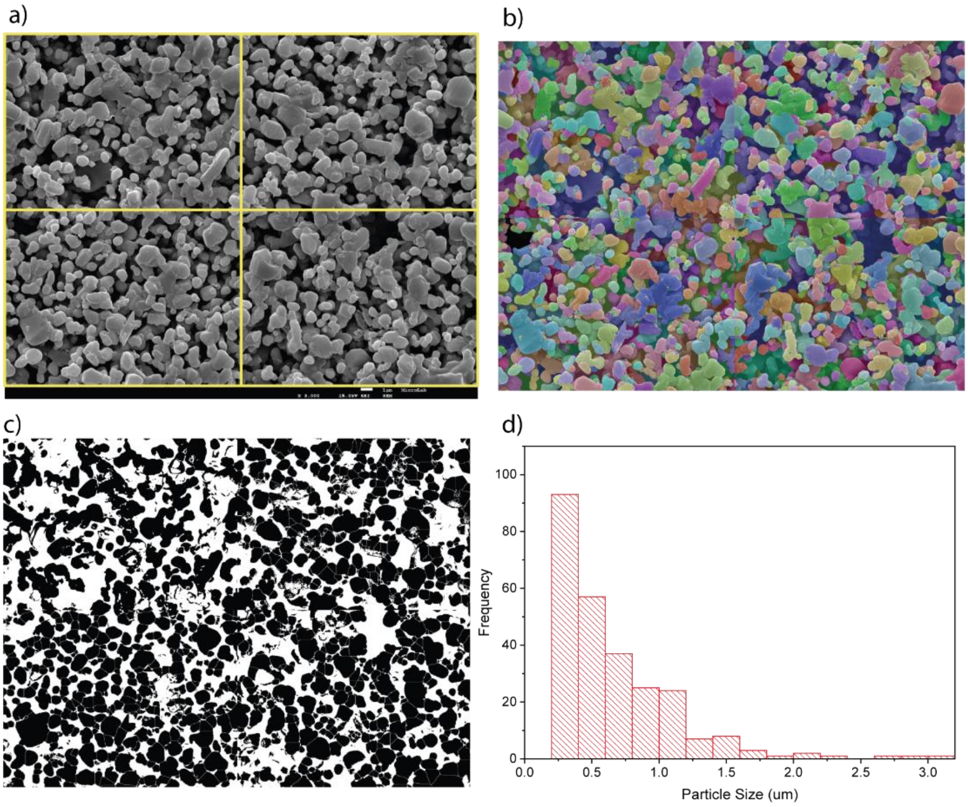

Figure S4. (**a**) Division of the micrograph (**b**) Morphological segmentation (**c**) Threshold adjustment (**d**) Normal distribution obtained with the values of particle size.

S4. Calculation of the pore density and percentage porosity of the membranes.

$$Pore\ density\ \left(\frac{1}{m^2}\right) = \frac{\#\ pores}{A_{mem}} \quad \text{(S1)}$$

$$Porosity\ (\%) = \frac{A_{pores}}{A_{mem}} \times 100 \quad \text{(S2)}$$

Where A_{mem} is the area of image analyzed and A_{pores} is the area occupied by the pores on the image.

S5. Calculation of the percentage of rejection (Equation (S3)) for both total coliforms and *E. coli.* was calculated.

$$\%\ Rejection = 100 \times \left(1 - \frac{C_p}{C_f}\right) \quad \text{(S3)}$$

Where, C_P = concentration of the respective bacteria in the permeate and C_f = concentrations of the same bacteria on the feed.

The percentage of treatment provided by the combined adsorption/retention and photocatalytic process was determined with a mass balance calculating the number of bacteria in the feed, permeate and retentate (Equation (S4)).

$$\%\ \text{Treatment} = 100 - (\frac{\left(\frac{\text{MPN}_\text{R}}{100} \times \text{V}_\text{R}\right) \times 100}{\left(\frac{\text{MPN}_\text{F}}{100} \times \text{V}_\text{F}\right) - \left(\frac{\text{MPN}_\text{P}}{100} \times \text{V}_\text{P}\right)} \quad \text{(S4)}$$

Where MPN_F, MPN_P, MPN_R represent the MPN of bacteria in the respective stream (Feed, Permeate or Retentate) per 100 mL of sample and V_F, V_P, V_R correspond to the volume of the respective sample.

During operation, the permeate flux was also calculated (Equation (S5)).

$$J = \frac{V_p}{A_m \times t} \quad \text{(S5)}$$

Where J is the permeate flux (L/m^2·h), Vp is the volume of sample permeated (L), A_m is the surface area of the membrane system (m^2) and t is the time required for the permeation (h).

S6. Defects found in the membranes after thermal treatment.

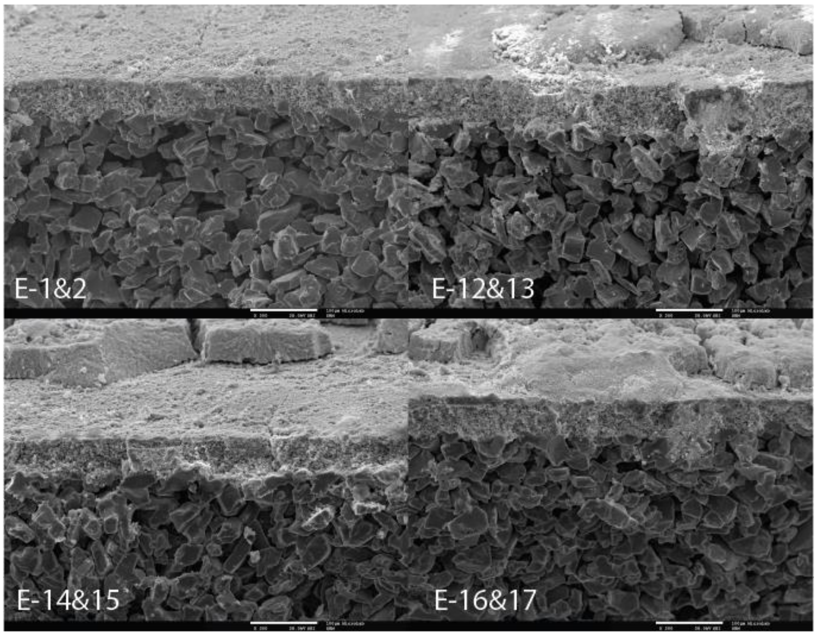

Figure S5. Cross section of the membranes modified and thermally treated.
